# Building New Jerusalem

**Published:** 1918-09-28

**Authors:** 


### "BUILDING NEW JERUSALEM."

### A Great Ideal to be Attained.

One wonders what meaning Blake himself attached to those often-quoted lines in which he prophesied the building of a *Civitas Dei* "in England's green and pleasant land," with a passionate hope. At that time the forces which have tended to turn our manufacturing towns into something more like Dante's City of Dis were already at work. However, the condition of human progress is always that the plodding toil of the practical worker shall translate the vision of the seer into terms of common life. In "Applied Christianity"* Mr. E. J. Smith finds a remedy for evils which this generation has only begun to see in the lurid light of war, and he shows us the beginnings, at least, of that application in the municipal efforts at betterment in his own town of Bradford. He believes, and shows cause for believing, that the work will be done less by the efforts of the Churches, which are too often hindered by attaching importance to organisations rather than the work to be accomplished through them, than by the diffusion of the spirit of the Master, "greatest Seer of Visions and greatest Democrat," among the rank and file of citizens. If we will replace—go on replacing—competition by co-operation, the building will progress as the laying of each course prepares for the next. The city which resolves that every man and woman and child of its population shall have a chance of leading a clean, wholesome, and happy life, that vice, drink, disease, child-torture, and resources squandered in misery shall be abolished, can effect its purposes. But undoubtedly the demand made upon the brain power, energy, and devotion of the administrators of such vast undertakings as are not merely prophesied, but in many cases shown to be in progress in this book, is immense. All the more welcome are the author's wise words upon membership of civic councils and committees. He recognises a great danger in the frequent co-option of persons for the very reason that they represent special interests, whereas knowledge and service accredited by the electors should be the only passport to authority. This rule, he rightly adds, should apply to women also. The fact that women are needed on boards of all kinds, and indeed are legally required in many cases, should lead to the qualifying of many more by service and not to the mere co-option of some important lady. That municipal service must lack "the human touch" of voluntary work the author somewhat indignantly denies, and surely with justice. No doubt the world in the past has owed much to the men and women whose originality of thought and courageous initiative have led them to devise, and to carry out, all manner of redemptive work for their fellows. There is also naturally a danger that organised and officialised work may become stagnant and bureaucratic. "Constant vigilance is the price of liberty," and in any community to cease to go forward is to slide back; the remedy lies in the constant re-kindling of the torch of enthusiasm as the older members of administrative bodies make way for younger energies.

The city of Bradford certainly serves well as a ground for experiment, being one in which 11,000 married women of child-bearing age, apart from widows, are employed in industry, and where actually 75 per cent. of the working classes still live in those back-to-back dwellings of which the erection was made illegal in 1909. Housing, therefore, is an acute problem, too serious to depend merely on private enterprise. To its present condition must be referred much of the ill-health of children, the misconduct of boys and girls, the overstrain and the slatternliness of women, the drunkenness of men. Cure cannot be found merely in pulling down and rebuilding on costly city sites, even if the L.G.B.'s maximum of twelve houses to the acre is not reached. Rather in the open country, linked up by modes of cheap and rapid transit, villages can be founded by municipal enterprise, on land "cheaper than linoleum," and houses so built that the decencies of life might be enjoyed, while the working mother is not worn out by hopeless toil. The communal laundry and kitchen and such amenities of life as the school, library, recreation-room, and playing field can find room in such places. The author gives the acreage of land in the United Kingdom as 77,000,000, and the number of dwellings at 9,000,000, adding that 483,000 persons live in one-roomed houses, 4,429,000 in those of three rooms. Statistics have frequently shown how much heavier is the incidence of mortality in over-crowded places in Bradford; it is 25 per 1,000 in one-and two-roomed houses, 12.4 in four-roomed ones, and there is as much common sense as eloquence in Mr. Smith's plea that expenditure on decent housing would eventually save millions of money. The vast expenditure on sanatoria—proud though Bradford has every reason to be of its Bierley Hall and other fine institutions—is another proof of our continued preference for curative to preventive measures, for dispensaries and hospitals rather than kitchens and clean milk supplies.

Mr. Smith frankly urges endowment of motherhood at the rate of five shillings per child of every married woman, and offers a touching argument in the recorded experience that frequently the very fact of the attention paid and care given to her child at an infant clinic arouses mother-love in a weary and bedraggled woman. As a help towards the heavy cost, he would tax not bachelors only, but childless married couples. His figures are extremely interesting with regard to the 2d. rate levied for the Health Committee's Maternity and Infant Welfare work, costing the town £13,000 a year, apart, of course, from the half-expenses grant of the L.G.B. When we are told that some mothers have living infants for the first time and others are able at last to give their babe its proper food since meals were given, the whisper that such work is "pauperising" dies. At the same time the careful calculation of incomes, and free

* *Race Regeneration.* By E. J. Smith. (London: P. S. King. 7s. 6d. net.)

(Continued on page 554.)

### BUILDING NEW JERUSALEM (continued from page 557).

gifts or graduated payments accordingly, seems admirably done, and great value attaches to the condition that each mother fed must be seen by a dentist and treated if necessary. The chapter on clean milk has the interesting statement that Lord Rhondda offered a monopoly of supply to the corporation which had previously applied for this, and received a refusal. It certainly provides a powerful argument for breast-feeding in the description of the short haul from mother to infant and the long haul from a distant cow, with fresh peril at every step of the process. Here, again, America outruns us. "Certified" milk there must not contain more than 10,000 bacteria to the cubic centimetre, or teaspoonful. A London dairy claiming special merit showed 800,000 to 8,000,000 in various samples. Poultry-keeping and vegetable-growing to meet the needs of the city's charitable institutions are undertaken by the Bradford Municipality. In fact, in a book full of interesting discussions of our most acute social problems and suggestions for their solution the point of greatest importance is the writer's conviction that the local authority should and must be the great reconstructive force. This being so, the multiplication of inspection and control by overlapping departments of central government would cease except in so far as proof of fitness must be required for Treasury grants-in-aid.

To education, in one special sense, we owe the rapid advance in efforts for national health, because it was the evident unfitness to receive education of 1,000,000 or more of school children which first compelled attention to the condition of the boy and girl. Then to the infant, to the mother, to the home, the question was pushed back. It is to education that we must look again, for without it all effort will be stultified. The slum landlord, for instance, is often no vulture, but a poor man or woman, kindhearted enough and sorely tried by tenants whom their slum life demoralises. To deliver them from the influences of yesterday and guide them to a nobler to-morrow is what we must mean by reconstruction. It is, in fact, to "build Jerusalem," and Mr. Smith deserves gratitude for his suggestions of practical steps to that end.

